# NCBI Orthologs: Public Resource and Scalable Method for Computing High-Precision Orthologs Across Eukaryotic Genomes

**DOI:** 10.1007/s00239-025-10268-2

**Published:** 2025-09-25

**Authors:** Dong-Ha Oh, Alexander Astashyn, Barbara Robbertse, Nuala A. O’leary, W. Ray Anderson, Laurie Breen, Eric Cox, Olga Ermolaeva, Robert Falk, Vichet Hem, J. Bradley Holmes, Patrick Masterson, Kelly M. McGarvey, Eyal Mozes, John P. Torcivia, Mirian T. N. Tsuchiya, Craig Wallin, Françoise Thibaud-Nissen, Terence D. Murphy, Vamsi K. Kodali

**Affiliations:** National Center for Biotechnology Information, National Library of Medicine, National Institutes of Health, Bethesda, MD 20894 USA

**Keywords:** Orthologs, NCBI, RefSeq, Comparative genomics resource, Synteny, Orthology, EGAP

## Abstract

Orthologs are fundamental for enabling comparative genomics analyses that further our understanding of eukaryotic biology. The unprecedented increase in the availability of high-quality eukaryotic genomes necessitates scalable and accurate methods for orthology inference. The National Center for Biotechnology Information (NCBI) developed “NCBI Orthologs”, a resource and a computational pipeline designed to meet this challenge within the NCBI RefSeq framework. This system integrates protein similarity, nucleotide alignment, and microsynteny to achieve high-precision ortholog assignments across diverse eukaryotes. The pipeline leverages high-quality RefSeq annotations and processes genomes individually, ensuring scalability. Resulting ortholog data, organized into gene-level anchored sets, enables propagation of functional annotation information and facilitates comparative genomics. Critically, these data are integrated into the NCBI Gene resource, providing users with access from various entry points. The NCBI Datasets resource provides an intuitive interface to explore orthologous relationships on the web and allows bulk data download via the web, command-line tools, and an API. We detail the methodology, including anchor species selection and the decision tree used to arrive at high-confidence one-to-one orthology relationships. NCBI Orthologs is a valuable resource for facilitating functional annotation efforts and enhancing our understanding of eukaryotic gene evolution.

**Supplementary Information:**

The online version contains supplementary material available at 10.1007/s00239-025-10268-2.

## Introduction

Community initiatives, coupled with advanced sequencing technologies and assembly algorithms, have led to a rapid expansion of high-quality, complete genomes across the eukaryotic tree of life (Rhie et al. 2021; Darwin Tree of Life Project 2022). The National Institutes of Health (NIH) Comparative Genomics Resource (CGR) project, spearheaded by the National Center for Biotechnology Information (NCBI) at the National Library of Medicine (NLM), has developed an NCBI genomics toolkit comprised of high-quality data and high-performance tools to maximize the utility and impact of these data across many fields of biology (Bornstein et al. 2023). A central component of this toolkit is uniformly high-quality annotation of eukaryotic genomes generated by the Eukaryotic Genome Annotation Pipeline (EGAP) and a network of orthologous genes that connect annotated genes from diverse organisms.

Gene function information, as determined through experimental and genetic analyses, is predominantly available for a limited set of established model organisms (Alliance of Genome Resources Consortium 2024) with significant community interest (Takeda and Shimada 2010; Klingler and Bucher 2022; Kocher and Kingwell 2024). For the vast majority of non-model species, gene functions are initially inferred based on their similarity to genes in model organisms. For example, information on gene functions and nomenclature provided by the research community for a model organism can be propagated to related non-model species within a clade based on orthologs (Gabaldon and Koonin 2013). This approach serves as a foundational starting point for exploring gene functions in non-model species.

Orthologs are genes that originated from a shared ancestor through a speciation event (Fitch 2000). Genomes from a pair of closely related species can be assumed to contain a set of one-to-one corresponding orthologs that share similar functions (Gabaldon and Koonin 2013). Orthologs that are conserved across a clade can serve as a resource to evaluate the completeness of the annotated gene set in a genome assembly (Manni et al. 2021; Nevers et al. 2025; Prieto-Banos et al. 2025). Orthologs organized as clustered groups and gene trees are essential for tracing the evolutionary trajectories of gene functions and their regulatory mechanisms. Through comparative analyses and phylogenetic profiling, researchers can gain insights into the evolutionary dynamics of gene functions, elucidating how they have adapted and diversified over time (Emms and Kelly 2019; Thomas et al. 2022; Majidian et al. 2025). This understanding not only informs evolutionary biology but also functional genomics and the study of related genes across species.

The accurate inference of orthologous relationships is a significant challenge. Various biological processes, including gene gain, loss, and duplication, introduce substantial complexities in the underlying data (Force et al. 1999). Furthermore, current computational methods for orthology inference, which largely rely on sequence comparisons, often struggle to scale effectively with the increasing volume of high-quality genomic data. While resources like Ensembl Compara (Herrero et al. 2016), OrthoMCL-DB (Fischer et al. 2011), OrthoDB (Tegenfeldt et al. 2025), PANTHER (Thomas et al. 2022), and OMA (Altenhoff et al. 2024a, b) provide valuable orthology data, many are relatively static, lagging behind the continuous updates to genome assemblies and gene model annotations driven by new evidence and expert curation. Moreover, existing repositories can lack the comprehensive integration with other relevant resources needed for thorough comparative analyses.

Most of the current methods of inferring orthology rely solely on protein sequences (Langschied et al. 2024), neglecting valuable evolutionary signals present at the genomic level, such as microsynteny of neighboring genes (Zhao and Schranz 2019; Lovell et al. 2022) and adjacent genome sequences (Kirilenko et al. 2023). This is often due to the lack of standardized genome annotation consistently available across all species under consideration. The issue is further exacerbated by the computational demands of incorporating such data, particularly as the genome assemblies and annotations are frequently updated. For over two decades, the RefSeq project at NCBI has provided evidence-based, iteratively refined genome annotations supported by expert curation for a wide range of eukaryotes (Goldfarb et al. 2025). The scope of RefSeq continues to expand to represent additional clades, including both model and non-model species. The RefSeq data, encompassing genomic, transcript, and protein sequences, along with structural annotations anchored by stable, unique identifiers, are disseminated across multiple NCBI resources. RefSeq’s comprehensive data spanning a broad taxonomic range establishes an ideal foundation for a repository of orthologous gene sets.

In this paper, we describe an ortholog calculation method that utilizes RefSeq gene model annotations to establish precise orthologous relationships. Our method first compares all protein-coding gene models between two RefSeq eukaryote genomes to identify homologous gene pairs. In a subsequent step, we compute similarities in exonic nucleotide sequences and combine them with microsynteny information to identify the best possible one-to-one ortholog pair. This multilevel approach of integrating sequence similarities at the protein and nucleotide level and consideration of microsynteny improves the resolution of ortholog calls, particularly among closely related paralogs. With the rapid expansion of eukaryote genomes (Rhie et al. 2021; Darwin Tree of Life Project 2022; Goldfarb et al. 2025), our approach provides a scalable solution to propagate information, such as gene nomenclature, from model species to all RefSeq eukaryote genomes with high-precision and organizes RefSeq genes in ortholog sets. Orthologs calculated for RefSeq genomes further play key roles in propagating functional and structural annotation. Comparative analyses based on orthologs can further improve gene models through curation efforts, which, in turn, improves ortholog inference.

## Methods

### Metrics for Genome-Based Ortholog Calculation

**Fig. 1:**
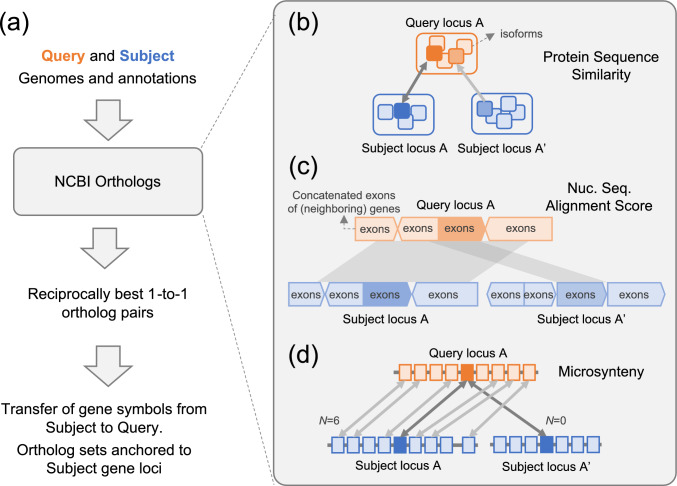
Schematic depiction of the NCBI Orthologs pipeline. **a** Orthologs between a pair of Query and Subject genomes were computed using the NCBI Orthologs pipeline **b–d** to generate reciprocally best one-to-one orthologs. Ortholog sets anchored on Subject gene loci are reported and used for propagating gene symbols from the Subject to Query gene loci. **b** All protein isoform sequences from Query genome were compared to those from Subject genome to identify the isoform pair exhibiting the highest protein similarity score to represent the Query and Subject locus pair. **c** To compute nucleotide sequence alignment scores, exonic sequences—including untranslated regions—were concatenated for each of the proteins from the isoform pair (panel b), further extended by 2 kb on both 5ʹ and 3ʹ ends using exonic sequence from adjacent genes, and aligned. **d** Microsynteny (N) was scored as the count of homologous gene pairs (indicated by bidirectional gray arrows) within a 20-locus neighborhood encompassing at most 10 adjacent loci on either side of the gene pair under consideration

To compute orthologs between a pair of genomes, designated as “query” and “subject”, the NCBI Orthologs pipeline utilized the following sets of input data for each genome: complete genome sequences, annotation information for all protein-coding genes, and the corresponding protein sequences (Fig. 1a). We considered a set of homologous gene pairs where the protein similarity scores (see below) were within 20% of the best score for either the query or subject gene. Our algorithm evaluated each candidate homologous gene pair between the query and subject genomes in the context of competing pairs. Each homolog gene pair (X, Y) was compared to all homolog pairs including either of the X or Y loci. This contextual evaluation was crucial, as true orthologs typically outperformed paralogous relationships across multiple metrics simultaneously. At every stage, we impose specific thresholds on the different metrics computed by the pipeline. These thresholds were empirically determined based on our experience in analyzing data of this nature during the development of EGAP and further refined as needed by reviewing data from multiple ortholog runs.

For each candidate pair, we extracted and evaluated the following metrics (Fig. 1b–d, Supplementary Fig. 1):

1. Protein Sequence similarity: Protein sequences from the query and subject genomes were compared in an all-versus-all manner using DIAMOND (‘-very-sensitive’ mode and reporting BLASTP-like alignment scores) (Buchfink et al. 2021). An earlier version of the pipeline used BLASTP (‘-evalue 0.001-word_size 6-threshold 21’) (Altschul et al. 1997), but we have recently switched to DIAMOND due to its superior performance while producing nearly identical results. Moving forward, the pipeline will use DIAMOND, though some public data were generated with the BLASTP pipeline. For each pair of query and subject genes, we selected the protein isoform pair exhibiting the highest protein similarity score. Raw alignment scores produced by BLASTP and DIAMOND don’t account for variations in protein lengths, presenting a key challenge in directly comparing scores between different protein pairs, as high score might simply reflect longer proteins rather than genuinely higher similarity. To overcome this, we adopted the traditional Jaccard index and modified it to suit the continuous nature of alignment scores. Our modified Jaccard index, calculated as “BLASTP_alignment_score / [sum_of_BLASTP_self_alignment_scores–BLASTP_alignment_score]”, normalizes the alignment score against the potential maximum similarity for that pair, allowing comparison across different combinations of protein isoforms for a given pair of query and subject genes.
2. Nucleotide-level conservation: For a given homologous gene pair represented by the best-scoring alignment between one protein isoform from each gene, we extracted sequences of all annotated exons, including untranslated regions, and concatenated them. These concatenated exonic sequences were then further extended to include additional 2 kb of flanking exonic sequences on both 5ʹ and 3ʹ ends, from adjacent genes. The resulting sequences were then aligned to each other using discontiguous-megablast (McGinnis and Madden 2004). Similar to the protein sequence comparison, a modified Jaccard index score was computed using the formula: “aligned_length/[sum_of_sequence_lengths_compared–aligned_length]”.
3. Microsynteny Conservation: Microsynteny was assessed by scoring the number of homologous gene pairs within a 20-locus window, encompassing at most 10 adjacent loci on either side of the gene pair under consideration.

### Selecting Orthologs Among Competing Homolog Pairs

Orthologs were identified by examining all homologous gene pairs involving the query and subject genes using an algorithm (detailed below and represented by pseudocode in Supplementary Fig. 1a) that relies on the metrics computed in the preceding section.

When microsynteny score is non-zero for the candidate pair, the algorithm determined the pair as orthologs if any of these conditions were met:

- The candidate pair had a non-zero microsynteny score and a protein similarity score greater than or equal to competing homolog pairs that had no microsynteny support.
- The candidate pair had a microsynteny score of at least 2, while competing pairs had no microsynteny support.
- The candidate pair’s microsynteny score exceeded that of competing pairs, its nucleotide alignment score was greater than or equal to any of the competing pairs, and its protein similarity exceeded all other competing homolog pairs by at least 5% and scored the highest for either the query or subject.
- The candidate pair’s microsynteny score exceeded that of competing pairs by at least 2, and its nucleotide alignment score was greater than or equal to any of the competing homolog pairs.

For candidates pairs with a microsynteny score of zero, stricter criteria were applied, requiring all of the following:

- No competing pair had microsynteny support.
- The protein alignment covered more than 50% of the longer protein and more than 90% of the shorter protein.
- Both protein similarity and nucleotide sequence alignment scores exceeded those of competing pairs by at least 5%.
- The protein similarity score was the highest for either the query or subject.

This dual-strategy approach enabled the algorithm to identify orthologs with high precision even in complex genomic contexts with multiple paralogs. The microsynteny component was particularly valuable for resolving cases where sequence-based metrics alone would be inconclusive, while the stringent criteria for cases without microsynteny ensured that only well-supported orthology assignments were accepted. See Supplementary Fig. 1b–d for examples to determine the orthologs based on the protein similarity, nucleotide sequence alignment, and microsynteny scores.

### Evaluation of NCBI Orthologs

To assess the performance of the NCBI Orthologs pipeline, we utilized the Quest for Orthologs (QfO) Orthology Benchmarking Service (https://orthology.benchmarkservice.org) (Nevers et al. 2022; Altenhoff et al. 2024a, b). The orthology research community established common QfO reference proteome datasets to facilitate comparative evaluations of different methodologies. Given that the “2020_04” dataset allows for the broadest comparison with existing methods, we selected this dataset as our benchmark. The QfO datasets consist of reference proteomes sourced from UniProt, which inherently lack the genome annotation information required by the NCBI Orthologs pipeline. Recognizing that a significant portion of these proteomes are derived from Ensembl annotations, we retrieved corresponding annotation data from Ensembl (Release 100) and integrated it with the proteomes. We then calculated orthologs for twelve vertebrates, using human, mouse and zebrafish as the anchor species, and for three arthropods using fruit fly as an anchor (Supplementary Table 1). To ensure compatibility, we mapped Ensembl gene identifiers to UniProtKB identifiers used in the QfO datasets. Genes that were orthologous to the same gene in the anchor genome were transitively considered as orthologs and added to the input submitted for QfO tests. We used the JSON reports of the QfO evaluation results to recreate plots in Supplementary Fig. 2. For public methods with multiple entries, we showed the best-performing entry as the representative (e.g. “sonicparanoid-mostsensitive” for SonicParanoid entries). For SwissTree challenge, we examined the raw results to identify false positive (FP) calls (see the note in Supplementary Data 1).

## Results

### Precise Calculation of Orthologs Anchored to Genes and Genomes at NCBI

Defining the taxonomic scope and the purpose for inferring orthologs is a crucial first step in developing an orthologs pipeline (Gabaldon and Koonin 2013). One of the important objectives of the RefSeq eukaryotic genome annotation process is to provide informative and consistent gene nomenclature. Using the network of orthologs, the RefSeq team aimed to leverage gene names from well-studied model organisms. For instance, organizations such as the HUGO Gene Nomenclature Committee (HGNC) (Seal et al. 2023) and FlyBase (Ozturk-Colak et al. 2024) have invested considerable effort in assigning informative gene names with input from the scientific community. The approach adopted by RefSeq imposes the following constraints on the data model: (1) the selection of an appropriate anchor species that has well-named genes, (2) a strict expectation of one-to-one orthologous relationships to ensure unambiguous gene naming, and (3) a stringent threshold for ortholog assignment to minimize the risk of mispairing paralogs between species—a strategy adopted to favor high-confidence calls, potentially at the expense of identifying all possible orthologs.

Gene duplication events frequently create paralogs within genomes, complicating the identification of true orthologous relationships between species. When a query genome contains N paralogs and a subject genome contains M paralogs, potentially N × M homologous pairs exist, though typically only a subset represents true one-to-one orthologs. To identify these orthologous pairs with high precision, we developed a multi-faceted algorithm that integrates multiple lines of evidence. A schematic diagram of our process to identify orthologs between two genomes is depicted in Fig. 1 and described in the Methods section. It is important to note that the unambiguous identification of a one-to-one ortholog pair is not always feasible, for example, due to recent gene duplication events. In such scenarios, the algorithm does not identify orthologs for any of the involved genes (Supplementary Fig. 1d).

### Evaluation of NCBI Orthologs

We evaluated the precision of our approach using the QfO challenge (Nevers et al. 2022). Considering our scope, we tested 1-to-1 best orthologs predicted between mouse and rat, human and other vertebrates, zebrafish and other fishes, and fruit fly and other arthropods included in the 2020 reference datasets (Supplementary Table 1). Prior to testing, the reference proteome datasets were mapped to genome annotations as detailed in the Methods section. We identified 193,307 ortholog pairs from direct comparison of the fifteen genome pairs. Genes that were orthologous to the same anchor gene were transitively inferred as orthologs to each other, adding 484,330 additional ortholog pairs to the input. In total, 677,637 ortholog pairs from thirteen vertebrate and four arthropod genomes were submitted for the evaluation using the QfO Orthology Benchmarking service.

QfO benchmark results exhibited a lower recall for the NCBI Orthologs method. We believe this was a result of the following reasons: (1) our pipeline strictly returns only one-to-one ortholog pairs and avoids any ambiguous calls, (2) we calculated orthologs for all species versus a select set of anchor species (e.g., human, zebrafish, and fruit fly) followed by transitive inference between non-anchor species, and (3) we calculated orthologs for vertebrates and arthropods only. On the other hand, the precision scores for NCBI Orthologs were the best among all methods in the Gene Ontology (GO) and Enzyme Classification (EC) challenges offered by QfO (Supplementary Fig. 2a, b). This relatively high precision persisted even when the results of other methods were filtered to include only the same species pairs evaluated by our pipeline (Supplementary Fig. 2c–d). For the SwissTree challenge, where the precision was inferred as Positive Predictive Value (PPV) based on test results from multiple gene trees, the absence of data for gene trees outside of our defined taxonomic scope led to a low PPV. Despite this, an analysis of raw results revealed 537 true positive calls without any false positive calls across 15 gene trees that included taxa for which we submitted our predictions (Supplementary Data 1). Similarly, NCBI Orthologs made only one false positive call due to a chimeric gene model alongside 21,730 true positives in the VGNC challenge (Supplementary Fig. 2e and Supplementary Data 1). We were unable to run the Generalized Species Tree Discordance test likely due to the limited scope of species pairs included in our submitted prediction.

### NCBI Orthologs for RefSeq Metazoan Genomes

While our pipeline is theoretically capable of computing orthologs between any two RefSeq genomes, we prioritized orthologs shared with a model organism anchor, selected based on the taxonomic relevance, to facilitate the primary purpose of propagating gene names. Given the extensive curation and community support provided by the HGNC (Seal et al. 2023) and the broader research community, the human genome (GRCh38, NCBI *Homo sapiens* Annotation Release GCF_000001405.40-RS_2024_08) was an obvious choice as an initial anchor. We designated it as the primary anchor for all vertebrate lineages.

**Fig. 2:**
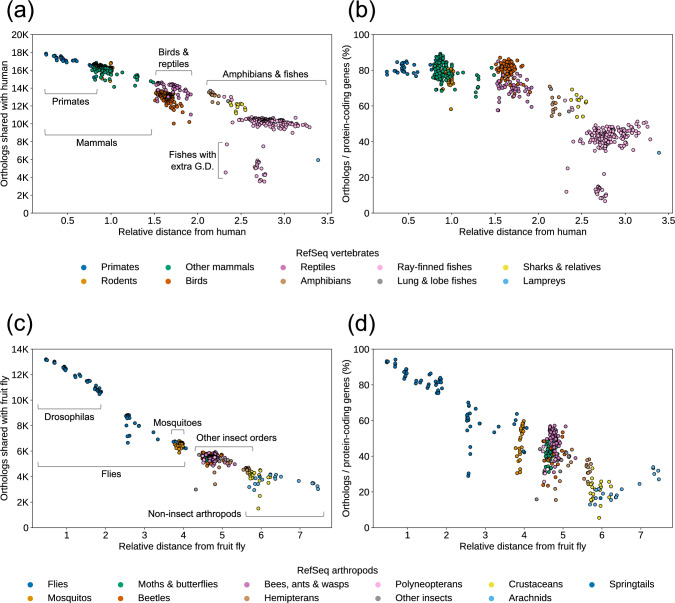
NCBI orthologs among RefSeq vertebrates and arthropods. **a**, **b** Total number and proportion of protein-coding genes with human orthologs for 577 vertebrate species. **c**, **d** Total number and proportion of protein-coding genes with fruit fly orthologs for 312 arthropod species. Ortholog counts and proportions are plotted against the relative evolutionary distance from the anchor genome. Evolutionary distance was inferred from the branch lengths of the RefSeq Metazoa tree (Fig. 3) and scaled such that the distance between human and mouse equals 1. See Supplementary Data 2 for data underlying these plots

The proportion of human orthologs among protein-coding genes in the query genome was affected by the distance from the human anchor as well as the gene content and genome duplication histories of the clade (Fig. 2a and b). Among primates, our pipeline identified between 15,196 and 17,869 orthologous gene pairs with human, covering between 75.5% to 85.6% of total protein-coding genes in each query genome. As we extended our analysis to more distant taxa within vertebrates, the absolute number of ortholog calls has decreased (Fig. 2a). Despite the decrease in absolute counts, the proportion of protein-coding genes with identified orthologs remained substantial across various vertebrate clades (Fig. 2b). We reported orthologs for an average 79.4% (± 4.5%) of protein-coding genes in mammals and birds. The average proportion of orthologs was reduced to 69.9% (± 5.3%) in reptiles, largely due to their genomes containing more protein-coding genes compared to those of birds with similar evolutionary distances from human (Supplementary Data 2). For amphibians, the proportion of orthologs among protein-coding genes was reduced further (60.7 ± 4.9%), mirroring the increasing distance from the human anchor (Fig. 2b and Supplementary Data 2).

**Fig. 3:**
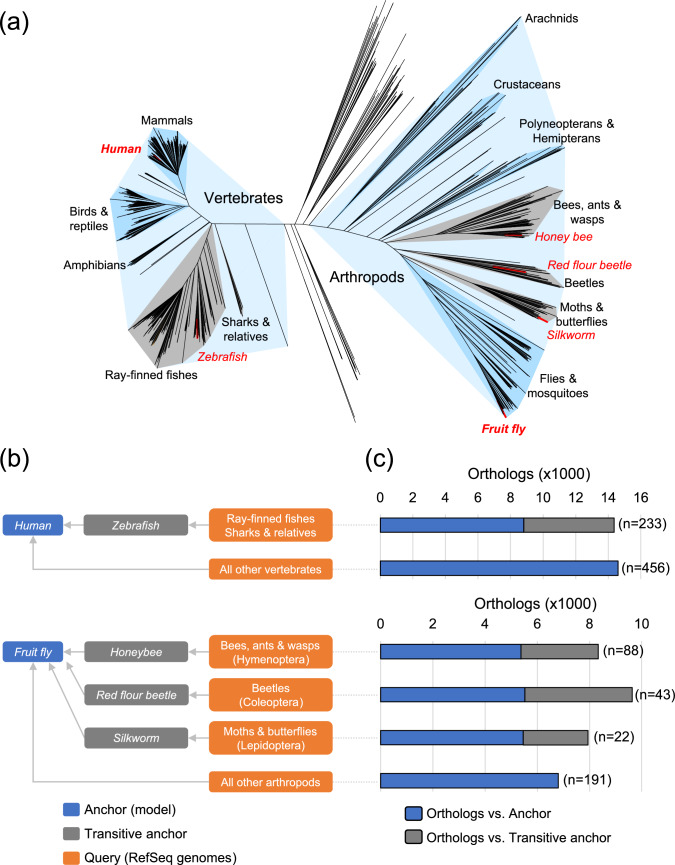
NCBI Orthologs anchor species and transitive orthology. **a** Phylogenetic tree of metazoan species represented in the RefSeq catalog. Species selected as ortholog anchors are shown in red text. Human and fruit fly serve as primary anchors for vertebrates and arthropods, respectively. Major clades within vertebrates and arthropods containing three or more species are shaded in darker blue. Clades large enough to benefit from a transitive anchor are shaded in gray, with the transitive anchor species indicated in red text. **b** The ortholog computation pipeline automatically selects an anchor species (blue) or a transitive anchor species (gray) based on the taxonomic grouping of the query species (orange). For a given query gene, if an ortholog was identified in a transitive anchor species, and that transitive anchor gene was also identified as an ortholog of a primary anchor gene, the query gene was transitively inferred as an ortholog of the primary anchor gene. **c** Average number of orthologs identified for RefSeq metazoan species within each clade shown in (**b**). When a transitive anchor was introduced, a significant number of orthologs were identified exclusively with the transitive anchor (gray bars). Orthologs identified with the primary anchor (blue bars) represent cases where the transitive anchor gene was also an ortholog of the primary anchor gene. The total number of query species in each clade (n) is indicated. See Supplementary Data 3 for data underlying these plots

The direct inference of orthologs between human and fish genomes presented a significant challenge. Due to the extensive history of gene duplications and ploidy events within fish lineages, both the absolute number and proportion of orthologs identified with human drastically decreased. For instance, the ray-finned fish clade includes a substantial number of RefSeq genomes (216 as of March 2025). Large distance from the human anchor (Fig. 3a) and ancient genome duplications (Taylor et al. 2003) resulted in a low coverage of orthologs among fishes (Fig. 2a and b). To mitigate this issue, we added the zebrafish reference genome (GRCz11, NCBI *Danio rerio* Annotation Release 106) as a transitive anchor to identify orthologs for all RefSeq genomes for ray-finned fish as well as sharks and relatives (Fig. 3b). When the zebrafish ortholog of a fish gene was also identified as an ortholog of a human gene, we considered the fish gene to be transitively an ortholog of the human gene (Fig. 3c, blue bar graph). Addition of the zebrafish transitive anchor identified orthologs among fish RefSeq genomes without a human ortholog, substantially increasing the ortholog coverage among fish genomes (Fig. 3c, gray bar graph).

We observed a similar pattern among orthologs shared by arthropod RefSeq genomes and the fruit fly (*Drosophila melanogaster*) reference genome (FlyBase Release 6.54) (Fig. 2c and d). For the arthropod clade, which covered a broader space in the RefSeq Metazoa tree than the Chordata and vertebrate clade (Fig. 3a), we used fruit fly as the model anchor (Fig. 3c). As RefSeq arthropod genomes expanded, clades that included a significant number of species that are evolutionarily distant from fruit fly emerged. These included the insect orders Hymenoptera, Coleoptera, and Lepidoptera. Similar to zebrafish for the fish clades, we used *Apis mellifera* (honeybee), *Tribolium castaneum* (red flour beetle), and *Bombyx mori* (silkworm) as transitive anchors for their respective orders to identify clade-specific orthologs (Fig. 3c, gray bar graph) in addition to orthologs shared with the fruit fly anchor (Fig. 3c, blue bar graph). Adding a clade-specific transitive anchor resulted in improved ortholog detection supported by stronger microsynteny signals (Supplementary Table 2). The numbers of orthologs shared with the model anchors or clade-specific transitive anchors for all vertebrate and arthropod RefSeq genomes as of March 2025 are summarized in Supplementary Data 3.

The ortholog inference process is tightly integrated into EGAP (Goldfarb et al. 2025). Upon examination of ortholog counts and microsynteny scores across multiple clades using different anchors (Fig. 3 and Supplementary Table 2), we identified one vertebrate (zebrafish) and three arthropods (honeybee, silkworm, and red flour beetle) to serve as transitive anchors in addition to human and fruit fly, which serve as the primary anchors. EGAP automatically chooses an appropriate anchor based on the taxonomy of the genome being annotated. When a transitive anchor was chosen, orthologs between the query genome and the primary anchor were inferred transitively based on the orthologs between the primary anchor and the transitive anchor. While the introduction of transitive anchors was necessary for effectively propagating names among genes that are specific to a clade, we note that the nature of orthologs are, unlike homologs, not necessarily transitive (Fitch 2000; Altenhoff et al. 2019). We extensively tested and confirmed that the query-to-primary anchor orthologs which were transitively inferred agreed with those calculated directly between the query and the primary anchor, with few exceptions (< 10 per query genome) involving mostly either a highly duplicated gene family, sub-optimal query, or transitive anchor gene models requiring manual curation.

### Ortholog and Nomenclature Assignment

The primary output of the ortholog computation pipeline is a comprehensive table which enumerates all query-subject protein pairs that pass the initial all-versus-all protein alignment step, providing an exhaustive set of metrics including protein and nucleotide alignment statistics and the count of neighbors exhibiting microsynteny (Supplementary Fig. 1b–d). Notably, the table also includes a column indicating whether each protein pair has been identified as orthologous.

Internally, these tables are loaded into an SQL database and used for gene naming and reporting purposes. All gene pairs identified as orthologs are consolidated into ortholog sets. Each ortholog set is represented by the anchor GeneID and consists of GeneIDs for all genes identified as orthologs to that anchor gene. NCBI GeneIDs are stable, numerical identifiers that are trackable across different annotation versions even when the underlying sequence data or associated metadata (such as gene names, symbols, aliases, and descriptions) undergo changes (Goldfarb et al. 2025).

In cases involving transitive orthology, orthologs are computed solely between the query genome and its most specific anchor species. For example, when bumblebee is the query genome being annotated, honeybee is automatically selected as the anchor species. The output data are then loaded into the database, where transitive orthologs to fruit fly are inferred for each bumblebee gene that has a corresponding honeybee ortholog. Bumblebee genes for which fruit fly orthologs are reported are subsequently added to the fruit fly ortholog sets, whereas the remaining bumblebee genes for which only honeybee orthologs are identified, are added to the honeybee ortholog sets.

In a final step, gene names are propagated from the ortholog anchor to all members of each ortholog set. This step is crucial, as it enables the assignment of informative gene names for many species, significantly enhancing the utility of RefSeq genome annotations. For instance, among the 33 *Drosophila* species annotated prior to 2023, the average number of protein-coding genes was 14,105. Our pipeline identified an average of 11,743 ortholog pairs per species. Initially, nearly all protein-coding genes were assigned placeholder symbols of the format LOC followed by the unique NCBI GeneID (for example, LOC108648959). However, leveraging orthology for gene symbol propagation significantly increased the number of genes with informative symbols to an average of 5,774 per species, with a maximum of 6,135. This effort, conducted in multiple phases with thorough expert curation at each step to ensure quality, has demonstrated the power of orthology in assigning meaningful nomenclature. Building on this success and increasing confidence in the assigned names, we have expanded this name propagation strategy to other insect species, resulting in over 1.38 million genes across nearly 350 insect species now possessing informative gene symbols.

Absent the orthologs pipeline, most genes in non-model organisms would lack informative names, as these organisms typically are not covered by dedicated nomenclature assignment groups. Nomenclature updates in anchor species are automatically propagated to all members of the ortholog set, and we have established mechanisms to prevent automatic updates for specific genes when deemed necessary. All gene names, as well as their historical versions which are included as aliases, are accessible in the NCBI Gene resource and across other NCBI products, such as Genome Data Viewer (Rangwala et al. 2021) and Comparative Genome Viewer (Rangwala et al. 2024), enabling users to browse and search using informative gene names.

In addition to nomenclature propagation, ortholog data are useful in identifying structural annotation issues. For instance, we systematically query ortholog sets to detect cases where orthologs for one or only a few species are missing. Such absences can stem from various reasons, including species-specific gene loss, gene duplication events leading to our pipeline’s failure to identify a single best ortholog, or an underlying issue in structural annotation. The RefSeq team of expert curators examine these flagged cases and take appropriate corrective actions. One example demonstrating this workflow is the “prune” gene (Supplementary Fig. 3). After observing the honeybee prune gene was unexpectedly missing from the fruit fly ortholog set (with anchor GeneID 31194), we investigated further. The full results table produced by the pipeline showed an alignment between honeybee gene LOC725150 (GeneID 725150) and the fruit fly gene “pn” (GeneID 31194). Upon reviewing additional data such as RNA-seq coverage, it became clear that the annotation of LOC725150 was indeed a chimera of two adjacent genes. To rectify this, we suppressed the existing transcript (XM_006568615.3) which encoded the chimeric protein (XP_006568678.1), created NM_001434528.1 to represent LOC725150, and created a new “Pn” gene (GeneID 138447619) with transcript NM_001434529.1 as depicted in Supplementary Fig. 3.

### NCBI Orthologs for RefSeq Protozoan Human Pathogens

NCBI employs the Eukaryotic Annotation Propagation Pipeline to propagate structural annotations submitted by users to RefSeq genomes of selected protozoa, fungi and a small number of other eukaryotic organisms of significant interest to the research community (Goldfarb et al. 2025). Protozoa, within NCBI, are informally defined as eukaryotic organisms excluding metazoa, viridiplantae, and fungi. At the time of this study, the RefSeq collection comprised 129 protozoan species, including several medically significant organisms implicated in human diseases such as malaria, African sleeping sickness, and Chagas disease. To facilitate comparative genomics analyses of these organisms, we employed our pipeline to identify orthologous gene pairs among them.

**Fig. 4:**
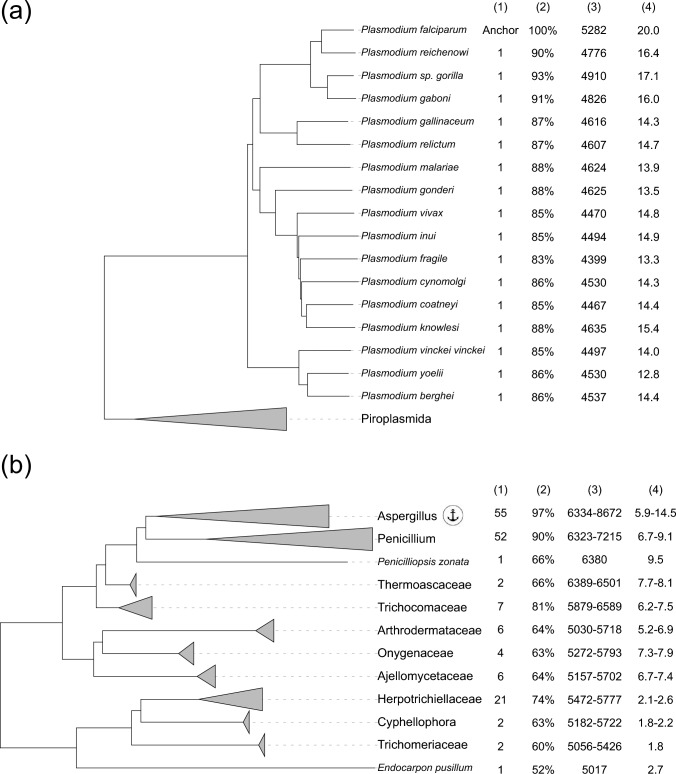
Statistics of *Plasmodium* and *Eurotiomycetes* orthologs. **a**, **b** Excerpts from the protozoa and fungi phylogenetic trees juxtaposed to tables depicting, for each species or clade: (1) the number of distinct genome assemblies included within each branch of the tree, (2) the percentage of the respective anchor species’ (*Plasmodium falciparum 3D7* or *Aspergillus fumigatus Af293*) protein-coding genes for which orthologs have been identified, (3) the range of orthologs computed for each species (**a**) or all species within the clade (**b**), and (4) the range of average neighbor counts

For the initial set of computations, we selected species from the genera *Plasmodium*, *Trypanosoma*, *Leishmania*, and *Toxoplasma*. These genera collectively represented nearly half of the protozoan species within the RefSeq collection at the time, are all medically relevant, and have substantial support from the research community, exemplified by resources such as VEuPathDB (Alvarez-Jarreta et al. 2024). Direct ortholog inference using a single anchor species proved impractical due to the large taxonomic distance between these genera and the limited number of ortholog pairs that could be identified. Consequently, we selected *Plasmodium falciparum 3D7* (Fig. 4a), *Trypanosoma brucei brucei TREU927*, *Toxoplasma gondii ME49*, and *Leishmania major strain Friedlin* as individual anchor species for ortholog computation. The complete list of species analyzed and the corresponding number of identified ortholog pairs are presented in Supplementary Data 4. Our process identified an average of 5975 (*N* = 8), 7311 (*N* = 12), 4572 (*N* = 17), and 3266 (*N* = 8) ortholog pairs for the anchors *T. brucei*, *L. major*, *P. falciparum*, and *T. gondii*, respectively.

For *Plasmodium* and *Leishmania*, our pipeline identified orthologs for over 94% of the protein-coding genes in their respective anchor species. This high proportion is likely because we calculated orthologs for species within the same genus or subfamily as the anchors. Indeed, we were able to identify orthologs for 64.6% of *P. falciparum* and 69.6% of *L. major* protein-coding genes across all 17 and 12 species, respectively. On the other hand, only 26.3% of *T. brucei* protein-coding genes had orthologs across all 8 *Trypanosoma* species. Among the *Trypanosoma* genomes analyzed, the *T. cruzi* genome (GCF_000209065.1) notably featured an unusually large size (~ 90 Mbp) and protein-coding gene count (19,607) compared to the others (mean ~ 23.8Mbp and 9,768 genes). We hypothesize that the *T. cruzi* genome represents an unresolved diploid or a hybrid, leading to a larger proportion of highly similar paralogs (El-Sayed et al. 2005; Weatherly et al. 2009). Our algorithm, which avoids making ortholog calls when identification of an unambiguously pair is not feasible (as exemplified in Supplementary Fig. 1d), consequently identified fewer orthologs for *T. cruzi.* Excluding *T. cruzi*, the proportion of anchor genes with orthologs in all *Tryponosoma* species increased to 54.9%. Among the protozoan parasites tested, *Toxoplasma gondii* served as the anchor for the most diverse group of species. *T. gondii* shared the highest number of orthologs with its two closest species, *Neospora caninum* and *Besnoitia besnoiti* (Supplementary Data 4).

### NCBI Orthologs for Selected RefSeq Fungi

Similar to protozoa, the RefSeq collection of Fungi relies on high-quality genomes with user-submitted gene model annotations available in GenBank. The genomes of organisms with significant research interest are selected based on criteria described in (Alvarez-Jarreta et al. 2024; Goldfarb et al. 2025) and processed through the Eukaryotic Annotation Propagation Pipeline to generate RefSeq genome assemblies with gene model annotations in a standardized format. While ortholog computation has not yet been integrated into this pipeline, the value of orthologous relationships in enhancing gene understanding is undeniable. The availability of orthologous relationships to genes from multiple distinct genomes significantly increases the likelihood of assigning informative protein names.

Consequently, we computed orthologous relationships for 370 fungal species, using the following taxa as comparison anchors within their respective taxonomic classes or orders: *Aspergillus fumigatus strain Af293* assembly GCF_000002655.1 (Eurotiomycetes, 159 taxa), *Saccharomyces cerevisiae strain S288C* assembly GCF_000146045.2 (Saccharomycetes, 31 taxa), *Candida albicans strain SC5314* assembly GCF_000182965.3 (Serinales, 35 taxa), *Fusarium oxysporum strain Fo47* assembly GCF_013085055.1 (Sordariomycetes, 133 taxa), and *Rhizopus microsporus strain ATCC 52813* assembly GCF_002708625.1 (Mucorales, 12 taxa). These anchor species were selected due to their association with extensive research over decades, their status as the taxa with the most downloaded assembly counts, and their association with widely studied strains. Our analysis identified an average of 6556 (*N* = 159), 4208 (*N* = 31), 4683 (*N* = 35), 7236 (*N* = 133), and 5983 (*N* = 12) ortholog pairs for the anchors *A. fumigatus, S. cerevisiae, C. albicans, F. oxysporum*, and *R. microsporus*, respectively (Supplementary Data 5).

*Aspergillus fumigatus* is a thermotolerant species and the primary causal agent of invasive aspergillosis (Beer et al. 2018). This species belongs to a genus which includes multiple toxin producing species which can infect a variety of eukaryotes (animal and plant) but also species important to the food, biotechnology and drug industries (Navale et al. 2021). As expected, the highest percentage of ortholog assignments for *A. fumigatus* was in the *Aspergillus* clade (97%, 55 taxa) and 7% less in the sister clade *Penicillium* (52 taxa) (Fig. 4). The highest number of orthologs (7441–8672) and average ortholog neighbor count (9.6–14.5) were assigned to species in the same subgenus, *Fumigati* (Supplementary Data 5).

*Saccharomyces cerevisiae*, commonly known as brewer’s yeast, has been used in baking, brewing and winemaking since ancient times. This yeast is an important model organism for molecular and cell biology research with a highly curated reference assembly and consistent genetic nomenclature (Wong et al. 2023). The highest percentage (92%) of ortholog assignments from *S. cerevisiae* was to species in its own genus (*Saccharomyces* clade, 4 taxa) and dropped down to 57% in the most distant and early diverging clade (Ascoideales, 2 taxa) (Supplementary Fig. 4a). The yeasts *S. cerevisiae* and *C. albicans* are common members of the healthy human mycobiome (Nash et al. 2017). However, *C. albican*s belongs to a different class (Pichiomycetes) of yeasts and is a member of the order Serinales (the CTG codon codes for serine instead of leucine) which contain several human pathogens and has the potential to cause candidiasis under certain conditions such as an immunocompromised state, dysbiosis or damage to the muco-intestinal barrier (Talapko et al. 2021). This taxonomic order also includes *Candidozyma auris*, a species which rapidly emerged as a serious threat in hospital acquired infections and quickly gained multidrug resistance across the globe (Lockhart et al. 2017). 96% of *C. albicans* proteins were assigned orthologs to proteins of species in the *Candida* (senso stricto) clade whereas the percentage dropped to 78% for the *Candidozyma* clade (Supplementary Fig. 4b) containing *C. auris* and related taxa which were previously known as *Candida* species but recently reclassified (Liu et al. 2024).

*Fusarium oxysporum* is a soil-borne fungus, mostly known as an economically important plant pathogen of various crops. However, *F. oxysporum* and other species in the genus are also opportunistic human pathogens, producing toxins and capable of localized or life-threatening disseminated infections depending on the status of an individual’s immune system (Al-Hatmi et al. 2016). The percentage of ortholog assignments of *F. oxysporum* proteins was 97% in the *Fusarium* clade containing 20 taxa (Supplementary Fig. 4c). The average orthologous neighbor count ranged from 5.5 -12.3 for taxa in the same order (Hypocreales) but as the microsynteny deteriorated dropped to 2.3–4.3 for taxa in other orders and more distant clades (Supplementary Data 5).

*Rhizopus microsporus* is a plant pathogen causing rot in various crops, spoilage of fresh and manufactured food, yet is also used in the production of fermented foods (Nout and Kiers 2005; Napo et al. 2025). In addition, *R. microsporus* is a healthcare-associated opportunistic pathogen and mainly affects predisposed individuals (Bowers et al. 2020). Interestingly, it has been found that a bacterial endosymbiont helps the fungus to evade the clearance by human macrophages (Itabangi et al. 2022). The percentage of anchor proteins with assigned orthologs to other species and clades in the Mucorales were comparatively low to that observed for taxonomic groups in the Ascomycota. Percentages ranged from 75% assigned as orthologs in the *Mucoraceae* family clade (not the family that *R. microsporus* belong to) to 48% in *Phycomyces blakesleeanus* and with low average ortholog neighbor counts (1–2.7) across taxa in the order (Supplementary Fig. 4d). Genetic diversity, low sampling and lack of high quality and annotated assemblies likely contributed to this outcome. Currently no annotated chromosome or complete level assembly is publicly available, and the anchor assembly will likely be updated or switched in future as data quality improve.

### Data Access

NCBI Ortholog data, including sequence and metadata, can be accessed through NCBI Datasets (O’Leary et al. 2024). This resource provides both a web interface and programmatic tools to facilitate intuitive and user-friendly downloading of ortholog data.

**Web Access**: To explore ortholog data via the NCBI website, users can begin by visiting the NCBI homepage www.ncbi.nlm.nih.gov and entering a species name along with a gene symbol or description in the search bar (e.g. Homo sapiens ACE2). If the queried gene is part of an NCBI Ortholog set, its gene-specific knowledge panel will include an “Orthologs” button, which links directly to the corresponding ortholog page. This page allows users to browse ortholog sets by taxonomy, download associated sequences and metadata, and optionally align selected sequences using the COBALT multiple sequence alignment tool (Papadopoulos and Agarwala 2007). Additionally, the page provides links to view the gene’s genomic context using the NCBI Genome Data Viewer (Rangwala et al. 2021).

**Fig. 5:**
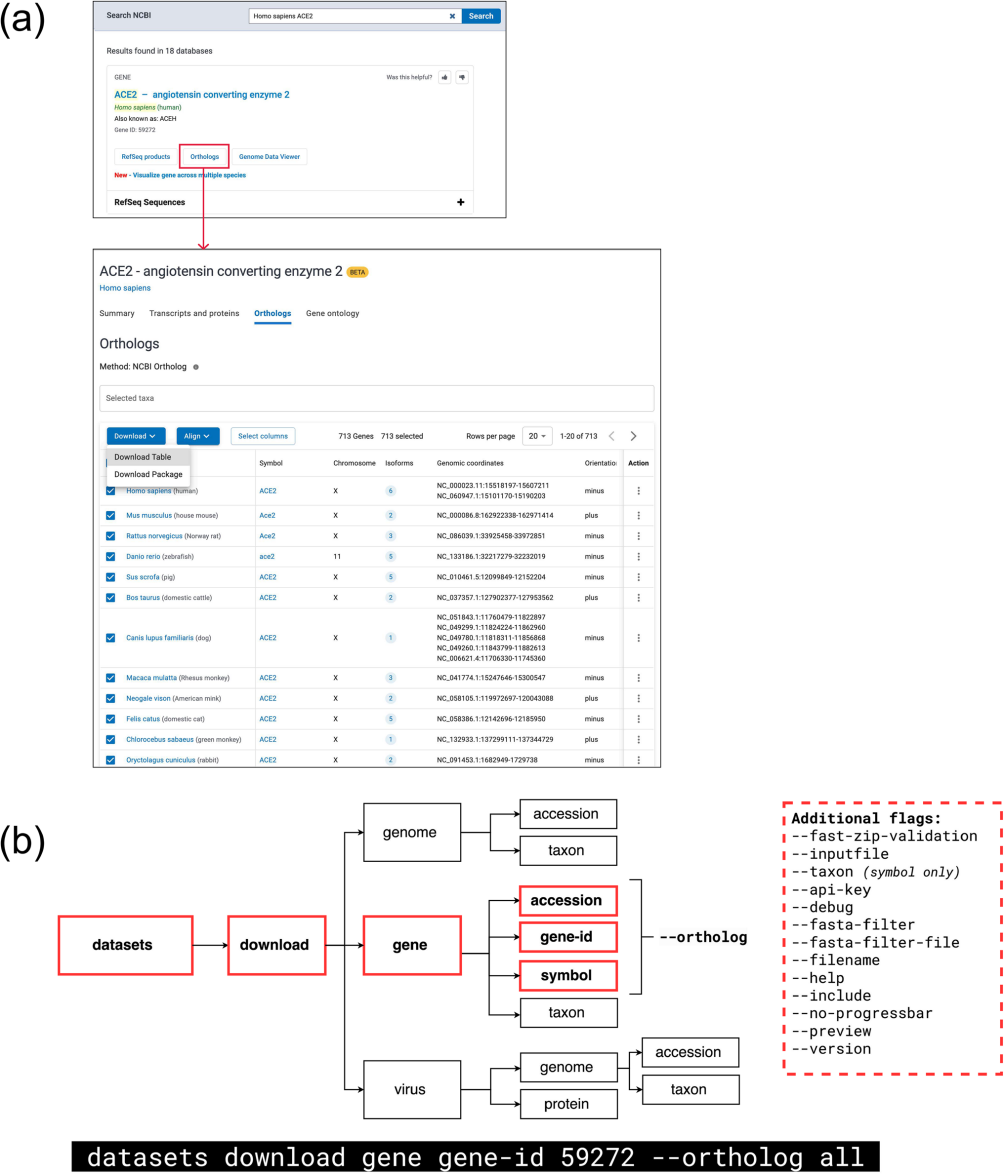
Accessing NCBI Orthologs **a** To find NCBI orthologs, visit the NCBI website and enter a species name and gene symbol in the search box. The resulting knowledge panel includes a link to the ortholog page, which features a table where users can browse and view members of the ortholog group. A download button enables the creation of a custom package containing sequences and metadata. **b** The NCBI Datasets command-line tool offers programmatic access to ortholog group data. This image illustrates the process using the ‘datasets download gene’ command combined with the ‘ortholog’ flag, which retrieves all one-to-one orthologs for the specified gene

**Programmatic Access:** NCBI Ortholog data can be accessed programmatically using the NCBI Datasets command-line tools https://www.ncbi.nlm.nih.gov/datasets/docs/v2/command-line-tools/download-and-install/. The datasets tool enables users to view metadata for genes included in the ortholog set or download a gene data package containing sequence and metadata for one or more ortholog sets. Data retrieval can be specified using a gene symbol and taxon name, GeneID, or accession number. The --ortholog flag allows users to download all available orthologs (--ortholog all) or a filtered subset based on taxonomy (e.g., --ortholog mammals). The resulting gene data package includes both sequence data and metadata, delivered in a ZIP archive. Users can customize the download to include specific sequence types such as FASTA files for genes, transcripts, proteins, untranslated regions (UTRs), and coding sequences (CDSs). Metadata and annotations for genes, transcripts, and proteins are provided as data reports in JSON Lines (.jsonl) format. The dataformat tool, which complements datasets, can be used to generate metadata tables (.tsv) from these data reports for easier analysis. When downloading multiple ortholog sets, each gene entry in the data_report.jsonl file includes a “gene_groups” section. This section specifies the ortholog methodology (e.g., NCBI Orthologs) and the identifier for the ortholog set, which corresponds to the GeneID of the anchor organism. This information can be used to organize and categorize ortholog sets. For detailed guidance on retrieving ortholog data using the Datasets command-line tools, consult the NCBI Datasets how-to guide, available at: https://www.ncbi.nlm.nih.gov/datasets/docs/v2/how-tos/genes/download-ortholog-data-package/. Additionally, a comprehensive file containing all gene ortholog pairs is available via FTP at: https://ftp.ncbi.nlm.nih.gov/gene/DATA/gene_orthologs.gz (see Fig. 5).

## Discussion

Here, we describe the methodology employed by NCBI for the computation and reporting of orthologous relationships, a fundamental process seamlessly integrated into every EGAP annotation run to facilitate accurate and consistent gene nomenclature assignment. As genome assemblies and evidence data improve, and NCBI continues to refine EGAP, gene model annotations are routinely updated to incorporate these advancements.

The development of NCBI’s ortholog pipeline and its associated data model has been primarily driven by the need to propagate gene names. This specific application has shaped the adoption of several core principles that underpin our approach. Firstly, we adhere to strict one-to-one ortholog pairings to ensure unambiguous gene name propagation. Secondly, the ability to scale the process to many hundreds of genomes is essential. Given the high volume of eukaryotic genomes annotated by NCBI annually, the pipeline is engineered to process individual genomes efficiently, obviating the computationally intensive need to recompute orthologous groupings across the entire dataset with the addition of new genomes and annotations. Thirdly, orthologs are anchored at the gene level, utilizing stable, unique identifiers that are independent of specific genome assembly versions. This decoupling is essential for accommodating frequent annotation updates, driven by both improvements to the EGAP pipeline and the availability of new genomic or transcriptomic data, thereby ensuring data consistency and minimizing disruptions to user workflows. Finally, the one-to-one nature of our ortholog calls facilitated the adoption of a simplified “ortholog sets” data model, where all genes within a set are considered orthologous to each other. The assignment of the anchor species’ GeneID as the identifier for each ortholog set further streamlines data management and eliminates the necessity for generating distinct unique identifiers for each ortholog group.

These guiding principles not only effectively serve the immediate needs for gene name propagation within NCBI but also present a contrasting approach to the methodologies employed by other similar resources. For instance, graph-based methods, which consider all-by-all relationships, often necessitate a complete recalculation of the entire ortholog dataset whenever new genomes are incorporated. While strategies like constructing a “core” phylogenetic tree and subsequently attaching genes from newly sequenced genomes can mitigate some of these computational burdens, they still represent a significant undertaking. The NCBI Orthologs pipeline distinguishes itself by integrating protein sequence similarities with comprehensive gene annotation information, including the context of neighboring exonic regions and microsynteny, to achieve a high degree of accuracy in ortholog inference. The reliance on complete annotation information, encompassing precise genomic locations of genes, transcripts, and exons, can be a considerable challenge for many individual research groups. However, EGAP’s capacity to generate consistent, high-quality annotations across a multitude of eukaryotic genomes, coupled with a standardized data model for representing genomic annotation, uniquely positions NCBI to perform such analyses and produce ortholog outputs of substantial value to the scientific community.

The approach adopted by NCBI Orthologs has inherent limitations. As described in the Results section, our algorithm is designed to conservatively exclude ambiguous ortholog calls, meaning if a single, best pair cannot be unambiguously identified, no ortholog call is made for the involved genes. We recognize that this approach does not represent paralogous relationships or other complex homology scenarios resulting from intricate evolutionary events. Consequently, a given ortholog set might lack species representation not only due to gene loss but also when certain gene duplication events lead to ambiguous ortholog calls. Conversely, if a one-to-one call is made despite a gene duplication event, nomenclature from the anchor species is propagated to only that single orthologous paralog in the query species. The remaining paralog(s) will then receive generic names, potentially obscuring the existence of additional related genes from users. This can fail to meet the needs of some users who are accustomed to one-to-many and many-to-many orthologous relationships. As we further develop this pipeline, we will carefully consider feedback from users and aim to meet their diverse needs. One potential approach is to make all results of the pipeline, including all homologous gene pairs examined along with all computed scores, publicly available to facilitate user-driven specialized analyses such as inferring one-to-many and many-to-many orthologous relationships.

While nomenclature propagation is a primary motivation for developing the NCBI orthologs pipeline, applying gene names across species can still be challenging. For instance, some genes may be named with organism or genome specific information that is not broadly applicable to other species such as phenotypes, tissues, or genomic locations, and gene clusters prone to lineage-specific changes in copy number such as histones and olfactory receptor genes are generally not amenable to orthology-based naming. Similarly, the *Drosophila melanogaster* nomenclature, which utilizes symbols of the format “CG” followed by a number for many genes, is not particularly descriptive, and propagating these to all arthropod orthologs would be of little benefit to users.

Recent additions of insect anchor species (*Apis mellifera*, *Bombyx mori*, and *Tribolium castaneum*) demonstrate a commitment to improving within-clade orthology representation. The selection of these anchors was based on careful analysis of RefSeq trees (Goldfarb et al. 2025) and ortholog computation results. We continue to monitor the RefSeq collection to identify additional transitive anchors to incorporate more clade-specific orthologs. For example, as of March 2025, RefSeq included non-insect arthropods such as 26 crustaceans and 21 arachnids. Our initial assessments indicated that the current sampling of genomes within these clades was insufficient to identify an anchor capable of adding a substantial number of clade-specific orthologs. For the time being, we report orthologs and propagate gene names using the model organism fruit fly as the anchor. While this approach limits the number of identified orthologs to a few thousand (Fig. 2c, d), these data still provide valuable resources for exploring gene functions, conservation, and evolution among non-model genomes.

While we continue to add anchors, this may still not serve the needs of all users, particularly those who want to compute orthologs between a specific pair of genomes outside our established anchor system. To address this, we intend to develop a standalone pipeline that users can run independently for more specialized needs. Ultimately, the NCBI Orthologs resource will continue to expand in parallel with the growth of genome data in RefSeq and its taxonomic scope.

## Supplementary Information

Below is the link to the electronic supplementary material.

Supplementary file1 (XLSX 414 KB)

Supplementary file2 (PDF 1039 KB)
