# Supplementary material for: NCBI Orthologs: Public Resource and Scalable Method for Computing High-Precision Orthologs Across Eukaryotic Genomes
Source: J Mol Evol. 2025 Sep 25;93(6):843–59. doi: 10.1007/s00239-025-10268-2 (PMC12756343; doi:10.1007/s00239-025-10268-2)
Supplement: Supplementary file 2 — Supplementary file2 (PDF 1039 KB) [file 239_2025_10268_MOESM2_ESM.pdf]

Supplementary Information

NCBI Orthologs: Public Resource and Scalable Method for Computing High-Precision Orthologs Across Eukaryotic Genomes

Dong-Ha Oh, Alexander Astashyn, Barbara Robbertse, Nuala A O'leary, W Ray Anderson, Laurie Breen, Eric Cox, Olga Ermolaeva, Robert Falk, Vichet Hem, J Bradley Holmes, Patrick Masterson, Kelly M McGarvey, Eyal Mozes, John Torcivia, Mirian T N Tsuchiya, Craig Wallin, Francoise Thibaud-Nissen, Terence D. Murphy, Vamsi K. Kodali*

National Center for Biotechnology Information, National Library of Medicine, National Institutes of Health, Bethesda, MD 20894, USA.

* To whom correspondence should be addressed: Email: kodalivk@ncbi.nlm.nih.gov

Supplementary Table 1 Orthologs calculated and submitted to the Quest for Orthologs (QfO) benchmarking service for evaluation

Anchor tax_id	Anchor organism name	Query tax_id	Query organism name	# Ortholog pairs
10090	*Mus musculus* (Mouse)	10116	*Rattus norvegicus* (Rat)	16,841
9606	*Homo sapiens* (Human)	9598	*Pan troglodytes* (Chimpanzee)	17,124
9606	*Homo sapiens* (Human)	9595	*Gorilla gorilla gorilla* (Western lowland gorilla)	16,809
9606	*Homo sapiens* (Human)	10090	*Mus musculus* (Mouse)	16,096
9606	*Homo sapiens* (Human)	9913	*Bos taurus* (Bovine)	15,783
9606	*Homo sapiens* (Human)	10116	*Rattus norvegicus* (Rat)	15,493
9606	*Homo sapiens* (Human)	9615	*Canis lupus familiaris* (Dog)	15,098
9606	*Homo sapiens* (Human)	13616	*Monodelphis domestica* (Gray short-tailed opossum)	13,964
9606	*Homo sapiens* (Human)	9031	*Gallus gallus* (Chicken)	12,329
9606	*Homo sapiens* (Human)	8364	*Xenopus tropicalis* (Western clawed frog)	12,262
7955	*Danio rerio* (Zebrafish)	8090	*Oryzias latipes* (Japanese rice fish)	13,860
7955	*Danio rerio* (Zebrafish)	7918	*Lepisosteus oculatus* (Spotted gar)	12,289
7227	*Drosophila melanogaster* (Fruit fly)	7165	*Anopheles gambiae* (African malaria mosquito)	6,216
7227	*Drosophila melanogaster* (Fruit fly)	7070	*Tribolium castaneum* (Red flour beetle)	5,685
7227	*Drosophila melanogaster* (Fruit fly)	6945	*Ixodes scapularis* (Black-legged tick)	3,458

Supplementary Table 2 Examples of orthologs gained by transitive anchor, together with the number of homologous neighbors (N).

RefSeq accession	Species name	Common name	# Protein-coding genes	# Orthologs vs. fruit fly (%)	Mean N vs. fruit fly	# Orthologs vs. honey bee (%)	Mean N vs. honey bee
GCF_003254395.2	*Apis mellifera*	honey bee	9,935	5,547 (55.8)	0.95	anchor	20.00
GCF_001442555.1	*Apis cerana*	Asiatic honeybee	10,719	5,255 (49.0)	0.92	**9,086 (84.8)**	**13.05**
GCF_910591885.1	*Bombus terrestris*	buff-tailed bumblebee	10,310	5,612 (54.4)	0.95	**8,844 (85.8)**	**12.43**
GCF_024542735.1	*Bombus huntii*		11,088	5,590 (50.4)	0.94	**8,814 (79.5)**	**12.42**
GCF_001652005.2	*Ceratina calcarata*		11,173	5,285 (47.3)	0.89	**8,632 (77.3)**	**6.49**
GCF_910589235.1	*Vespa crabro*	European hornet	10,204	5,512 (54.0)	0.92	**8,437 (82.7)**	**4.72**
GCF_905475345.1	*Vespula vulgaris*		9,996	5,530 (55.3)	0.92	**8,474 (84.8)**	**4.69**
GCF_016802725.1	*Solenopsis invicta*	red fire ant	14,790	5,514 (37.3)	0.92	**8,394 (56.8)**	**4.54**
GCF_013373865.1	*Monomorium pharaonis*	pharaoh ant	14,019	5,362 (38.2)	0.91	**8,367 (59.7)**	**4.47**
GCF_003672135.1	*Ooceraea biroi*	clonal raider ant	11,927	5,359 (44.9)	0.91	**8,312 (69.7)**	**4.36**
GCF_009193385.2	*Nasonia vitripennis*	jewel wasp	13,602	5,306 (39.0)	0.88	**7,730 (56.8)**	**2.91**
GCF_013357705.1	*Chelonus insularis*		10,548	5,458 (51.7)	0.92	**7,846 (74.4)**	**2.68**

(a)

```
"""
Simplified decision logic for classifying a pair of putative orthologs.

Input:

hp:  A candidate homolog pair with similarity scores and coverage metrics
top: A synthetic homolog pair constructed using maximum scores for each metric
     observed among all homolog pairs involving one of query or subject genes,
     except the homolog pair under consideration

Output:

Boolean value indicating if the pair should be classified as orthologs

Algorithm:
"""
@dataclass
class HomologPair:
    msy_sc         : int    # microsynteny score
    nuc_sc         : float  # nucleotide (exome-space) alignment score
    prot_sc        : float  # pairwise protein sequence similarity score
    prot_aln_len   : int    # length of the protein alignment
    q_cov          : float  # prot_aln_len / query protein length
    s_cov          : float  # prot_aln_len / subject protein length
    is_top_scoring_for_query_or_subject: bool
                            # whether prot_sc is maximal over other protein
                            # alignments involving these query and subject

def is_orthologs_pair(
    hp  : HomologPair, # Candidate pair of potentially orthologous genes.
    top : HomologPair  # Synthetic pair of orthologs with maximum possible
                       # scores for each metric among all homolog pairs
                       # involving either query or subject,
                       # excluding the current pair under consideration
) -> bool :
    is_nuc_maximal   = hp.nuc_sc >= top.nuc_sc
    is_prot_maximal  = hp.prot_sc >= top.prot_sc
    has_nuc_support  = 0.95 * hp.nuc_sc > top.nuc_sc
    has_prot_support = 0.95 * hp.prot_sc > top.prot_sc \
                      and hp.is_top_scoring_for_query_or_subject
    is_high_coverage = (
            min(hp.q_cov, hp.s_cov) > 0.5
        and max(hp.q_cov, hp.s_cov) > 0.9
    )

    has_microsynteny_support = (
           (hp.msy_sc > 0 and top.msy_sc == 0 and is_prot_maximal)
        or (hp.msy_sc > 1 and top.msy_sc == 0)
        or (hp.msy_sc > top.msy_sc + 1 and is_nuc_maximal)
        or (hp.msy_sc > top.msy_sc and is_nuc_maximal and has_prot_support)
    )

    # stricter criteria for a case without microsynteny support
    is_well_supported_outlier = (
            not hp.query.is_posible_pseudogene
        and not hp.subject.is_possible_pseudogene
        and top.msy_sc == 0
        and is_high_coverage
        and has_nuc_support
        and has_prot_support
    )

    return has_microsynteny_support or is_well_supported_outlier
```

(b)

	**Homolog pair 1**	Homolog pair 2	Homolog pair 3	Homolog pair 4
q_org	*Mus musculus*	*Mus musculus*	*Mus musculus*	*Mus musculus*
s_org	*Homo sapiens*	*Homo sapiens*	*Homo sapiens*	*Homo sapiens*
q_geneid (Gene symbol)	100504234 (Ccdc170)	100504234 (Ccdc170)	100504234 (Ccdc170)	80384 (Tex21)
s_geneid (Gene symbol)	80129 (CCDC170)	728047 (GOLGA8O)	643699 (GOLGA8N)	80129 (CCDC170)
q_multi	3	3	3	1
s_multi	2	5	5	2
q_prot	NP_001357921.1	NP_001357922.1	NP_001357922.1	XP_006516456.1
s_prot	NP_079335.2	XP_047288962.1	NP_001269423.1	XP_047275328.1
q_cov	0.9612	0.4789	0.5331	0.929
s_cov	0.9706	0.5137	0.5601	0.7469
prot_aln_len	694	318	354	484
prot_sc	0.506	0.0306	0.0325	0.0749
nuc_sc	0.1275	0	0	0
msy_sc	14	0	0	0
ortholog	T	F	F	F

(c)

	**Homolog pair 1**	Homolog pair 2	Homolog pair 3	Homolog pair 4
q_org	*Bos taurus*	*Bos taurus*	*Bos taurus*	*Bos taurus*
s_org	*Homo sapiens*	*Homo sapiens*	*Homo sapiens*	*Homo sapiens*
q_geneid (Gene symbol)	504248 (PRPS1L1)	504248 (PRPS1L1)	781227 (PRPS1)	781227 (PRPS1)
s_geneid (Gene symbol)	221823 (PRPS1L1)	5631 (PRPS1)	221823 (PRPS1L1)	5631 (PRPS1)
q_multi	3	3	3	3
s_multi	3	3	3	3
q_prot	NP_001095953.1	NP_001095953.1	NP_001039654.1	NP_001039654.1
s_prot	NP_787082.1	NP_002755.1	NP_787082.1	NP_002755.1
q_cov	0.9635	0.9635	1.0000	1.0000
s_cov	0.9969	0.9969	1.0000	1.0000
prot_aln_len	317	317	318	318
prot_sc	0.7304	0.8046	0.8936	1.0000
nuc_sc	0.1950	0.0948	0.0945	0.6113
msy_sc	15	0	0	11
ortholog	T	F	F	T

(d)

	**Homolog pair 1**	Homolog pair 2	Homolog pair 3	Homolog pair 4
q_org	*Bos taurus*	*Bos taurus*	*Bos taurus*	*Bos taurus*
s_org	*Homo sapiens*	*Homo sapiens*	*Homo sapiens*	*Homo sapiens*
q_geneid (Gene symbol)	617475 (LOC617475)	617475 (LOC617475)	617475 (LOC617475)	101902099 (F8A1)
s_geneid (Gene symbol)	8263 (F8A1)	474383 (F8A2)	474384 (F8A3)	8263 (F8A1)
q_multi	3	3	3	3
s_multi	2	2	2	2
q_prot	XP_002699745.1	XP_002699745.1	XP_002699745.1	XP_024844326.2
s_prot	NP_036283.2	NP_001007524.1	NP_001007525.1	NP_036283.2
q_cov	0.9946	0.9946	0.9946	0.9423
s_cov	0.9892	0.9892	0.9892	0.9677
prot_aln_len	367	367	367	359
prot_sc	0.7463	0.7463	0.7463	0.7099
nuc_sc	0.1144	0.1144	0.1144	0.3802
msy_sc	6	7	7	9
ortholog	F	F	F	T

Supplementary Fig. 1 Pseudocode to identify orthologs among homolog pairs and examples of metrics produced by the pipeline to illustrate the choice of orthologs. (**a**) shows the decision process to determine if a homolog pair is an ortholog. The homolog pair must meet the criteria for either the "has_microsynteny_support" or "is_well_supported_outlier", to qualify as an ortholog pair.

(**b**) This figure illustrates the evaluation of a mouse-human homolog pair ("Homolog pair 1") for orthology. The mouse gene (q_geneid = 100504234) had three homolog pairs (q_multi = 3) with human genes 80129, 728047, and 643699. Conversely, the human gene (s_geneid = 80129) was part of two homolog pairs (s_multi = 2) with mouse genes 100504234 and 80384. Homolog pair 1 (100504234, 80129) was assessed against all other homolog pairs involving either gene. Homolog pair 1 exhibited superior (bold and in blue color) protein similarity score (prot_sc), nucleotide sequence alignment score (nuc_sc), and microsynteny score (msy_sc) compared to the competing homolog pairs. Note that homologous gene pairs can be represented by different protein isoforms (q_prot and s_prot), which scored the highest protein similarity among all isoform pairs. Gene symbols were shown in parentheses together with the locus IDs. For homolog pairs sharing a member with the Homolog pair 1, scores superior and inferior compared to Homolog pair 1 were marked blue and red, respectively.

(**c**) The same table as (b) for a case where Homolog pair 1 (504248, 221823) between human and cattle was identified as an ortholog pair based on nucleotide sequence alignment score and strong microsynteny conservation despite lower protein similarity score compared to Homolog pair 2 (504248, 5631). Human gene 5631 (PRPS1) had another cattle homolog (781227), with the identical protein sequences and strong microsynteny signal, declared as an ortholog.

(**d**) In this example, the human genome included three paralogs F8A1/2/3 (coagulation factor VIII-associated 1-like 1/2/3), with F8A2 and F8A3 located adjacent to each other and F8A1 separated from F8A2/3 by several loci. All three encoded proteins with identical sequences. The cattle gene 617475 cannot be unambiguously paired with a human gene locus due to identical scores with F8A2 and F8A3 and lack of stronger microsynteny signal with F8A1. On the other hand, its paralog 101902099 was paired to human F8A1 as orthologs based on well conserved genes in the neighborhood (Homolog pair 4).

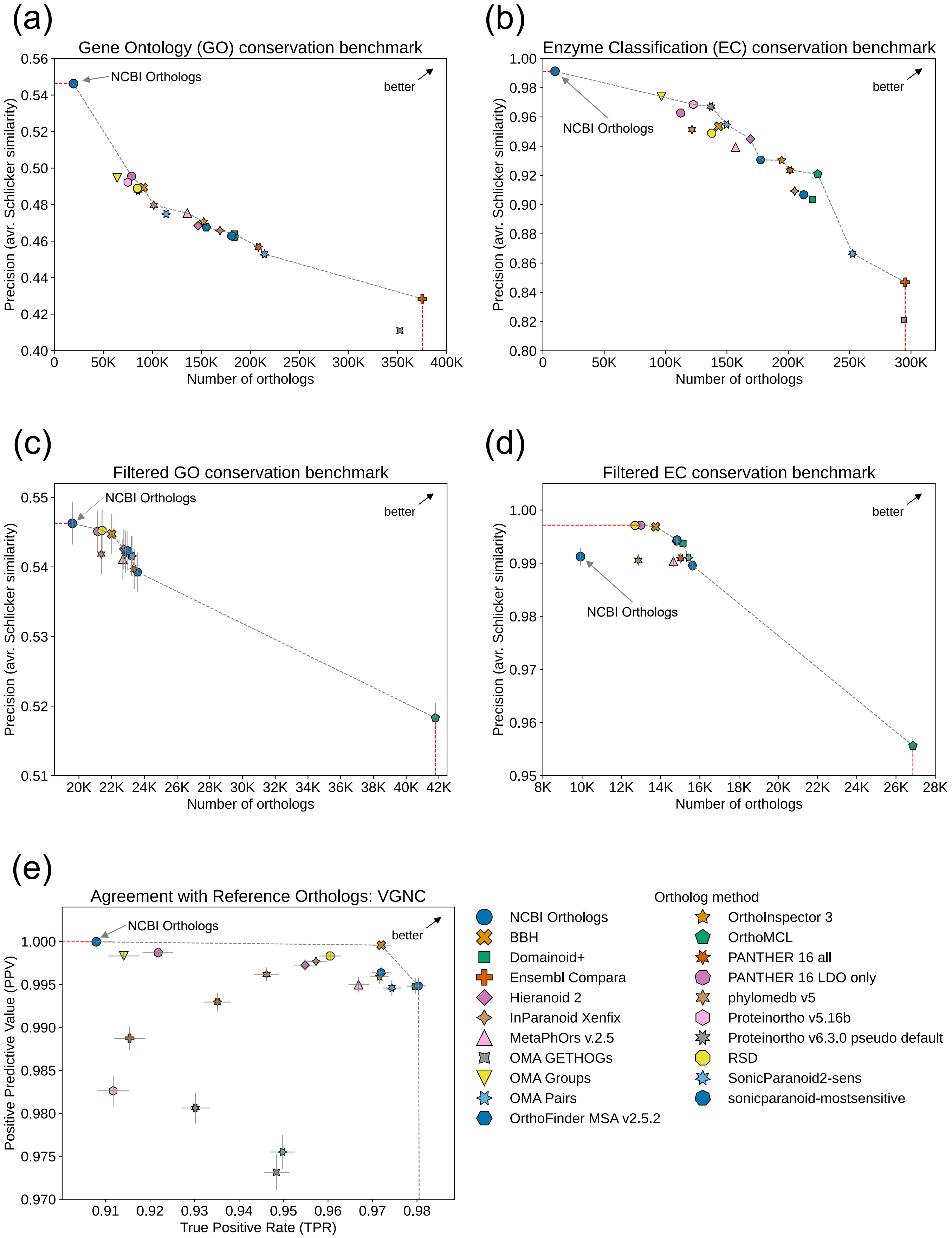

(a)
Gene Ontology (GO) conservation benchmark
NCBI Orthologs
better
Precision (avr. Schlicker similarity)
Number of orthologs
(b)
Enzyme Classification (EC) conservation benchmark
NCBI Orthologs
better
Precision (avr. Schlicker similarity)
Number of orthologs
(c)
Filtered GO conservation benchmark
NCBI Orthologs
better
Precision (avr. Schlicker similarity)
Number of orthologs
(d)
Filtered EC conservation benchmark
NCBI Orthologs
better
Precision (avr. Schlicker similarity)
Number of orthologs
(e)
Agreement with Reference Orthologs: VGNC
NCBI Orthologs
better
Positive Predictive Value (PPV)
True Positive Rate (TPR)
Ortholog method
NCBI Orthologs
BBH
Domainoid+
Ensembl Compara
Hieranoid 2
InParanoid Xenfix
MetaPhOrs v.2.5
OMA GETHOGs
OMA Groups
OMA Pairs
OrthoFinder MSA v2.5.2
OrthoInspector 3
OrthoMCL
PANTHER 16 all
PANTHER 16 LDO only
phylomedb v5
Proteinortho v5.16b
Proteinortho v6.3.0 pseudo default
RSD
SonicParanoid2-sens
sonicparanoid-mostsensitive

Supplementary Fig. 2 We evaluated NCBI Orthologs method using the Orthology Benchmarking service provided by the Quest for Orthologs (QfO) consortium (Altenhoff, Nevers, et al. 2024). Based on the April 2020 reference proteome datasets, we calculated orthologs between fishes versus zebrafish, other vertebrates versus human, and insects versus fruit fly as detailed in Methods and Supplementary Table 1. Orthologs of the same anchor gene were transitively inferred as orthologs among themselves. Publicly available results for twenty orthology methods were plotted together with NCBI Orthologs for the Gene Ontology (GO) conservation (**a, c**), Enzyme Classification (EC) conservation (**b, d**), and Agreement with Reference Orthologs: VGNC benchmark (**e**). Pareto fronts are indicated by dashed lines. In (**c**) and (**d**), GO and EC conservation benchmarks were run on a filtered set of predicted ortholog pairs. This set comprised data from all QfO 2020 methods whose ortholog pairs are available in simple tabular format. These ortholog pairs were then further subsetted to include only species pairs evaluated by our pipeline. This ensures a fair comparison and mitigates any bias from functional conservation varying with evolutionary distance. In (**e**), the Pareto front extended to the OrthoMCL data point (x=0.9828, y=0.4751) plotted below the y-axis range. See Nevers, et al. 2022 for details on how precision (y-axes), recall (x-axes), and their standard errors (error bars) were determined for each test. The raw QfO test results for the three tests plotted in (a—e), as well as the Agreement with Reference Gene Phylogenies: SwissTree test, are described in Supplementary Data 1.

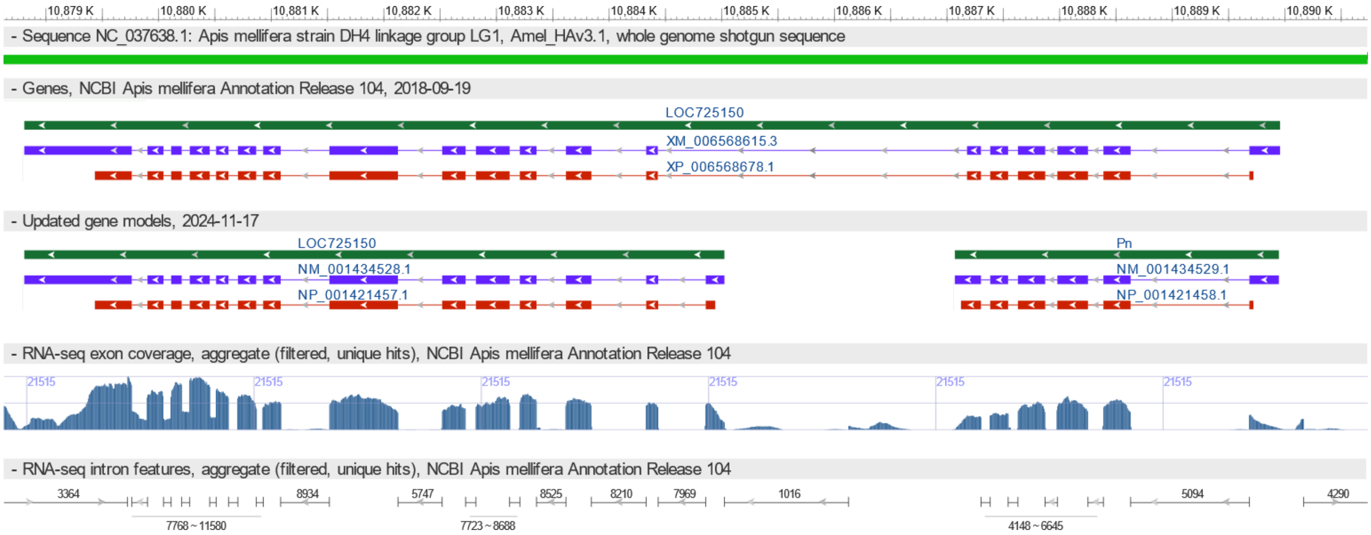

Supplementary Fig. 3 Curation of chimeric gene model in *Apis mellifera* annotation. Genome Workbench view of *Apis mellifera* chromosome LG1 (assembly GCF_003254395.2) comparing the original Annotation Release 104 annotation of gene LOC725150 with its currently public, updated version. Annotation Release 104 included a chimeric gene model for LOC725150, encompassing an adjacent gene. The RefSeq curation team resolved this by: (1) suppressing the existing chimeric transcript (XM_006568615.3); (2) creating a new transcript (NM_001434528.1) to accurately represent LOC725150; and (3) establishing a new Pn gene with its transcript (NM_001434529.1). Bottom tracks display RNA-seq evidence, including exon coverage data and reads spanning adjacent introns, supporting the corrected annotation.

(a)

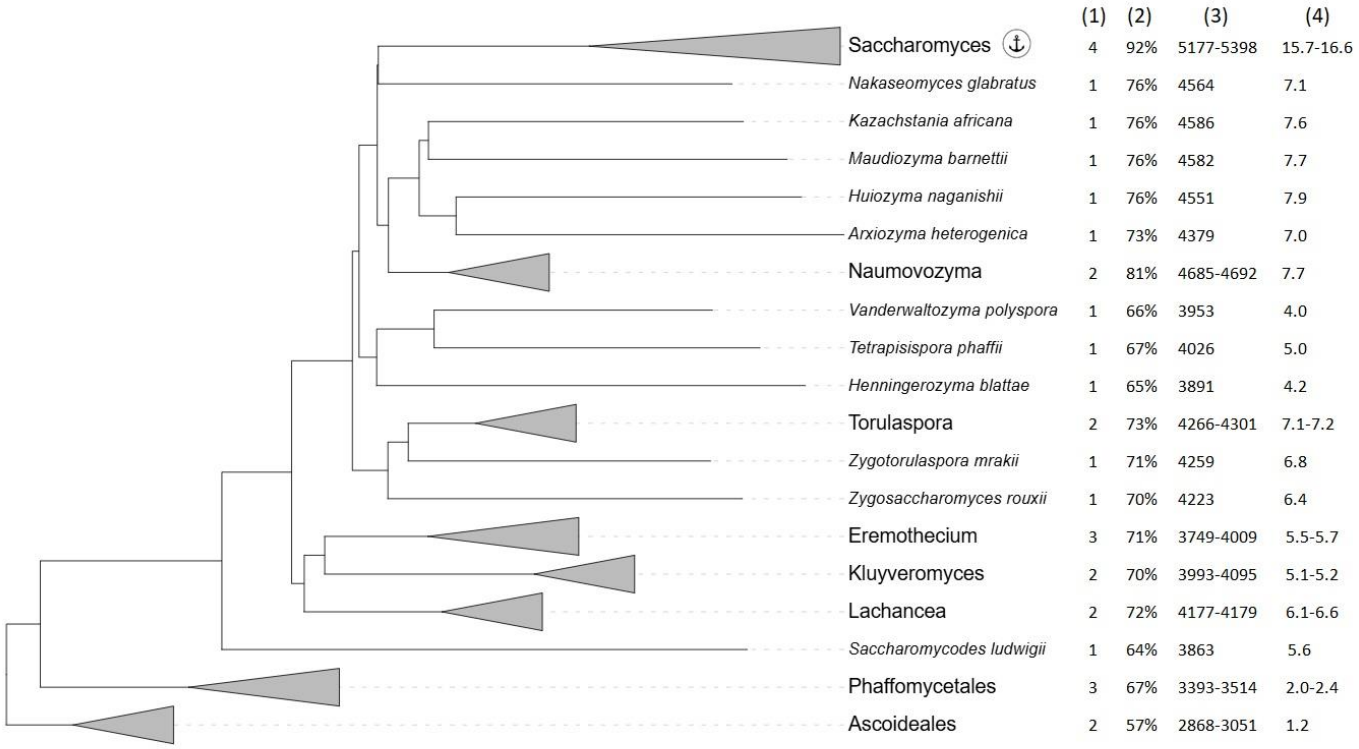

(1) (2) (3) (4)
Saccharomyces 4 92% 5177-5398 15.7-16.6
Nakaseomyces glabratus 1 76% 4564 7.1
Kazachstania africana 1 76% 4586 7.6
Maudiozyma barnettii 1 76% 4582 7.7
Huiozyma naganishii 1 76% 4551 7.9
Arxiozyma heterogenica 1 73% 4379 7.0
Naumovozyma 2 81% 4685-4692 7.7
Vanderwaltozyma polyspora 1 66% 3953 4.0
Tetrapisispora phaffii 1 67% 4026 5.0
Henningerozyma blattae 1 65% 3891 4.2
Torulaspora 2 73% 4266-4301 7.1-7.2
Zygotorulaspora mrakii 1 71% 4259 6.8
Zygosaccharomyces rouxii 1 70% 4223 6.4
Eremothecium 3 71% 3749-4009 5.5-5.7
Kluyveromyces 2 70% 3993-4095 5.1-5.2
Lachancea 2 72% 4177-4179 6.1-6.6
Saccharomycodes ludwigii 1 64% 3863 5.6
Phaffomycetales 3 67% 3393-3514 2.0-2.4
Ascoideales 2 57% 2868-3051 1.2

(b)

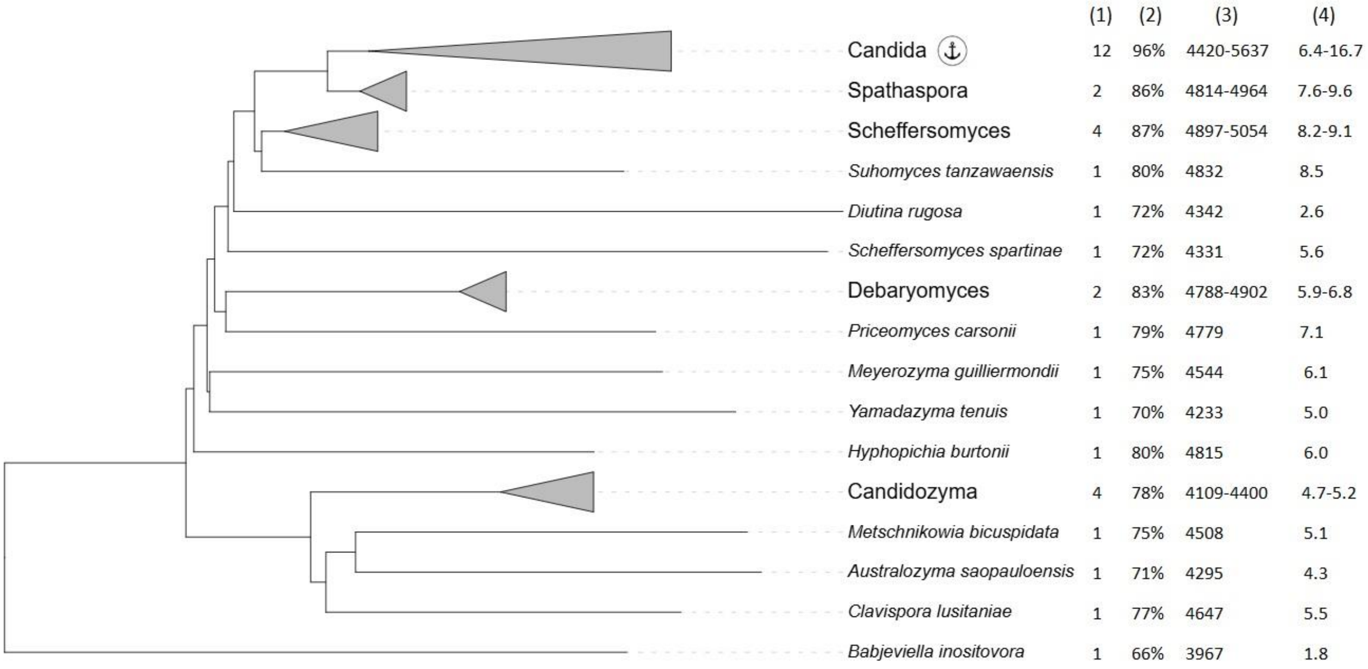

(1) (2) (3) (4)
Candida 12 96% 4420-5637 6.4-16.7
Spathaspora 2 86% 4814-4964 7.6-9.6
Scheffersomyces 4 87% 4897-5054 8.2-9.1
Suhomyces tanzawaensis 1 80% 4832 8.5
Diutina rugosa 1 72% 4342 2.6
Scheffersomyces spartinae 1 72% 4331 5.6
Debaryomyces 2 83% 4788-4902 5.9-6.8
Priceomyces carsonii 1 79% 4779 7.1
Meyerozyma guilliermondii 1 75% 4544 6.1
Yamadazyma tenuis 1 70% 4233 5.0
Hyphopichia burtonii 1 80% 4815 6.0
Candidozyma 4 78% 4109-4400 4.7-5.2
Metschnikowia bicuspidata 1 75% 4508 5.1
Australozyma saopauloensis 1 71% 4295 4.3
Clavispora lusitaniae 1 77% 4647 5.5
Babjeviella inositovora 1 66% 3967 1.8

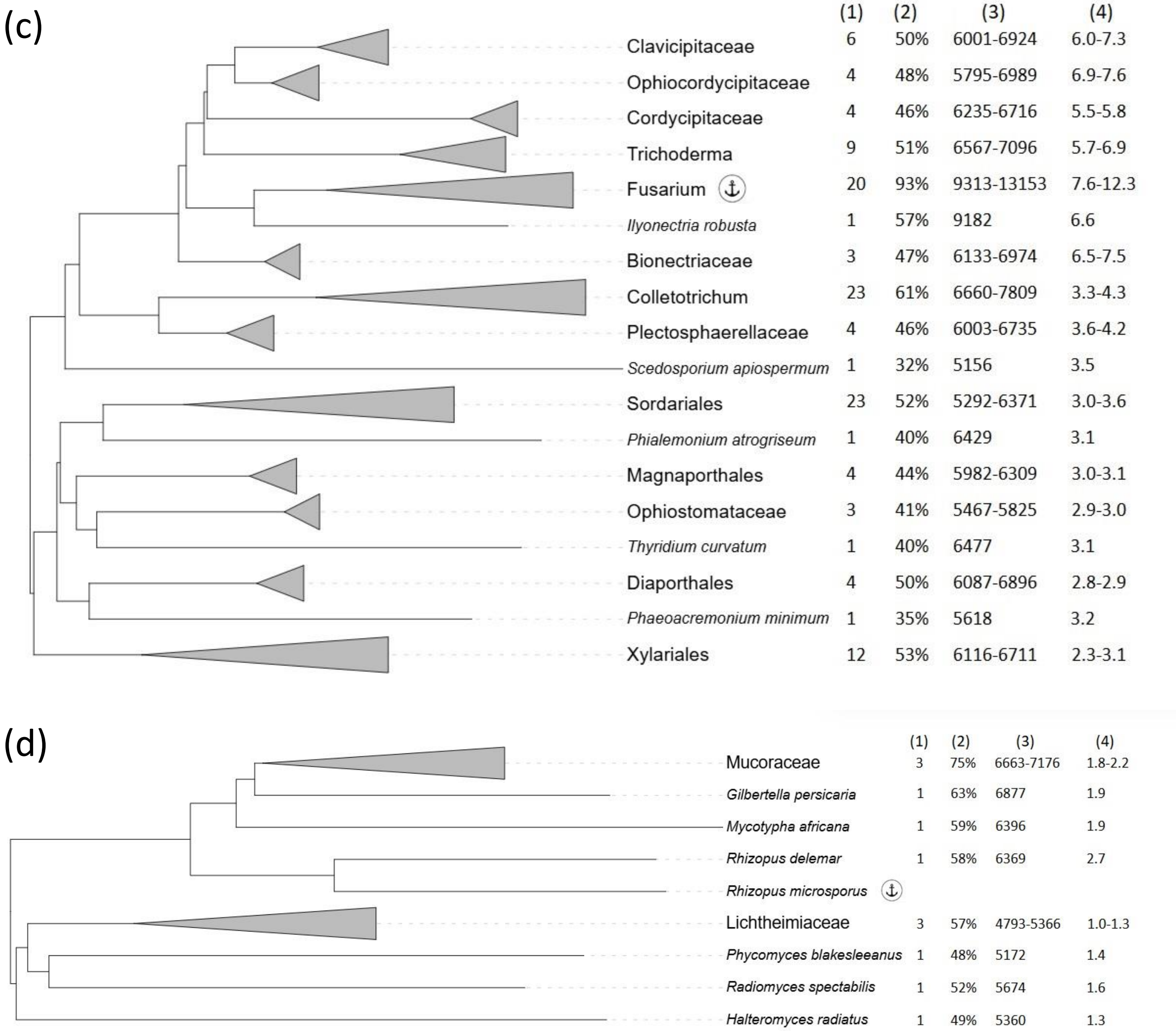

Supplementary Fig. 4 Excerpts from the Fungi genome-based protein distance tree showing relationships and ortholog statistics: (1) number of assemblies compared; (2) percentage of anchor proteins with orthologs; (3) ortholog count range (min—max) and (4) average orthologous neighbor count range (min—max), for the comparison anchor and RefSeq data in each taxonomic group: **(a)** *Saccharomyces cerevisiae* strain S288C proteins (16,202) on assembly GCF_000146045.2 compared within the *Saccharomycetes* (budding yeasts & others); **(b)** *Candida albicans* strain SC5314 proteins (6,030) on assembly GCF_000182965.3 compared within the *Serinales* (CUG-Ser1 clade in the class Pichiomycetes); **(c)** *Fusarium oxysporum* strain Fo47 proteins (16,202) on assembly GCF_013085055.1 compared within the *Sordariomycetes (a filamentous ascomycetes class)*; **(d)** *Rhizopus microsporus* strain ATCC 52813 proteins (10,888) on assembly GCF_002708625.1 compared within the *Mucorales (pin molds)*.
